# Universal Health Care System in India: An In-Depth Examination of the Ayushman Bharat Initiative

**DOI:** 10.7759/cureus.40733

**Published:** 2023-06-21

**Authors:** Harpreet Grewal, Pranjal Sharma, Gagandeep Dhillon, Ripudaman S Munjal, Ram K Verma, Rahul Kashyap

**Affiliations:** 1 Radiology, Florida State University College of Medicine, Pensacola, USA; 2 Nephrology, Northeast Ohio Medical University, Rootstown, USA; 3 Internal Medicine, University of Maryland Baltimore Washington Medical Center, Glen Burnie, USA; 4 Nephrology, Saint Joseph's Medical Center, Stockton, USA; 5 Sleep Medicine, Internal Medicine, and Obesity Medicine, Parkview Health System, Fort Wayne, USA; 6 Medicine, Drexel University College of Medicine, Philadelphia, USA; 7 Medicine, Harvard Medical School, Boston, USA; 8 Research, Global Remote Research Program, Saint Paul, USA; 9 Critical Care Medicine, Mayo Clinic, Rochester, USA

**Keywords:** universal health care for all, ayushman bharat pradhan mantri jan arogya yojana (ab pmjay), ayushman bharat, global healthcare systems, health services in india

## Abstract

This editorial provides an in-depth review of the Ayushman Bharat initiative, India's universal healthcare scheme, designed to address significant disparities in healthcare access and quality across the country. Following the structure of the healthcare system and socioeconomic trends, the manuscript assesses the reasons for the initiative's creation, its coverage, implementation strategies, role during the COVID-19 pandemic, auxiliary pilot programs, and challenges for future progress. It focuses on how the initiative has increased healthcare accessibility, financial protection, transformed the healthcare infrastructure, and provided relief during the COVID-19 crisis. Critical issues such as gaps between supply and demand, the need for increased government spending, and the challenges of access and quality in rural health centers are also discussed. We aim to raise awareness about the program's benefits among potential beneficiaries, which is a key to the initiative's success and a potential role model for equitable global healthcare.

## Editorial

Background

In response to the heterogeneity of healthcare availability and accessibility across rural and urban regions in India, the government introduced the Ayushman Bharat initiative, a flagship program aimed at achieving Universal Health Coverage (UHC) in alignment with the National Health Policy 2017 and Sustainable Development Goals (SDGs) of India. Launched in 2018, the scheme encompasses health and wellness centers (HWCs) and Pradhan Mantri Jan Arogya Yojana (PM-JAY) to address primary, secondary, and tertiary healthcare. This article comprehensively analyzes the Ayushman Bharat scheme, examining its impact on healthcare access, financial protection, and healthcare infrastructure. It has been a largely successful program, and this editorial highlights the program's key features, coverage, and implementation.

Why was the Ayushman Bharat initiative needed?

In recent years, India has witnessed substantial economic progress but continues to be categorized as a lower-middle-income country due to uneven socioeconomic and health factors. Over 20% of the population lives in poverty, and a demographic shift has occurred with 34% of the population aged 15-35, as per the census done in 2021 [1]. In addition, India is grappling with a "triple burden of disease" comprising persistent communicable diseases, a growing prevalence of non-communicable diseases, and injuries.

There are challenges for India's healthcare system to serve its 1.4 billion citizens adequately. The private sector handles around 70% of healthcare needs, but private providers are typically small, unregulated, and concentrated in urban areas, leaving underprivileged populations without proper care [1]. Public hospitals are overwhelmed and hindered by insufficient funding, a lack of skilled health professionals, inconsistent drug and equipment supplies, and often overcrowding with more patients than they can handle [1].

A significant factor contributing to these issues is India's ongoing underinvestment in public healthcare, with government spending on health remaining at approximately 2.1% of the Gross Domestic Product (GDP) [2]. As a result, out-of-pocket expenses make up 62% of India's total healthcare expenditure, exacerbating poverty and forcing nearly 60 million Indians back into poverty annually [2].

Earlier government-funded health insurance schemes, such as Rashtriya Swasthya Bima Yojna (RSBY), targeted secondary care hospitalization but overlooked primary healthcare. To address these challenges, the Indian Government launched the Ayushman Bharat initiative, aiming to reduce disease burden and hospitalization by transforming existing sub-centers and primary health centers into health and wellness centers. The program also introduced PM-JAY, the world's most extensive health insurance/assurance scheme, providing financial protection for various secondary and tertiary care hospitalizations for nearly 120 million impoverished families (550 million Indians) [2].

PM-JAY is entirely government-funded and seeks to decrease catastrophic out-of-pocket health expenses by enhancing access to quality healthcare for India's disadvantaged population. In the long term, PM-JAY aims to encourage the private sector to expand services in tier two and tier three cities and support public hospitals in prioritizing impoverished patients while generating additional revenue for infrastructure and service enhancement [1].

What does it cover?

Rural beneficiaries are covered if they meet at least one of six deprivation criteria (D1 to D5 and D7) and automatic inclusion criteria, such as destitution, manual scavenging, tribal groups, and bonded labor. The six deprivation criteria are detailed as follows: D1 - only one room with kucha walls and kucha roof; D2 - no adult member between ages 16 to 59; D3 - households with no adult male member between ages 16 to 59; D4 - disabled member and no able-bodied adult member; D5 - Scheduled Castes and Scheduled Tribes (SC/ST) households; and D7 - landless households deriving a major part of their income from manual casual labor [1].

Urban beneficiaries are eligible if they belong to one of the 11 occupational categories, including ragpickers, domestic workers, construction workers, and electricians [1].

PM-JAY aims to cover the bottom 40% of India's poor and vulnerable population, approximately 120 million households, using the Socioeconomic Caste Census 2011 (SECC 2011) database for rural and urban areas [3]. The SECC ranks households based on socioeconomic status and uses exclusion and inclusion criteria to identify automatically included and excluded households. PM-JAY utilizes this database to identify targeted beneficiary families. States with existing health insurance schemes and databases can use their own databases for PM-JAY, provided it is inclusive of SECC families. States can decide whether to participate in PMJAY. States are able to continue with their existing health insurance schemes with varying degrees of affiliation with PM-JAY (e.g., Andhra Pradesh, Karnataka, Kerala, Madhya Pradesh, Meghalaya, Punjab, Rajasthan, Tamil Nadu, and Telangana) [4]. The primary objectives of PM-JAY are to provide comprehensive coverage for catastrophic illnesses, reduce out-of-pocket expenses, enhance access to hospitalization, increase accessibility, and expand health insurance coverage. PM-JAY also aims to establish national standards for health assurance systems and provide national portability of care. At the implementation level, flexibility is granted to states, ensuring coverage for all families eligible as per the SECC data [3].

Implementation strategies

PM-JAY allows states to choose from three implementation models for their health insurance/assurance schemes: assurance/trust model, insurance model, and mixed model [1]. The assurance/trust model is commonly adopted, where the scheme is implemented by the State Health Agency (SHA) without involving an insurance company, and the government bears the financial risk, with a 60:40 ratio with the State government, in the majority of the states and Union Territories (UT) [1]. The insurance model involves selecting an insurance company through a competitive tendering process, and the company manages PM-JAY in the state, with the insurance company bearing the financial risk. The mixed model combines both assurance/trust and insurance models, which are used by states to provide more flexibility and allow convergence with the state scheme [1].

The administrative cost for each model is limited, and it varies between Category A (administrative cost not to exceed 20%) and Category B (administrative cost not to exceed 15%) states. If the claim settlement ratio (the ratio of claims paid out of the total number of claims received during a policy period) exceeds a certain percentage in any policy period, the excess amount is initially shared equally between the insurance company and the state government/Union Territory. Then, the central government shares the excess burden amount borne by the state government/Union Territory based on the sharing pattern ratio, usually in the 60:40 ratio for majority states and UTs [1]. Any additional amount beyond the central and state government's contribution must be borne by the insurance company. In the preauthorization procedure, once a medical treatment package is chosen for a patient, a request is sent to the insurer. The insurer must respond within six hours; if not, the request should be auto-approved per guidelines. The hospitals can register a patient up to five days after admission. Claim reimbursement should occur within fifteen days for claims within the same state and within thirty days for interstate claims. This is the maximum timeframe within which the insurer is expected to process and reimburse the claims, although some states, like Gujarat, have a more lenient time frame of forty-five days [5].

In order to ensure quality healthcare services delivery to beneficiaries under PM-JAY, hospitals must meet predetermined empanelment criteria. Two types of empanelment criteria exist, one for hospitals that provide non-specialized general medical and surgical care and another for hospitals with specialized clinical services. States oversee the empanelment process through State and District Empanelment Committees, which review and verify online applications from hospitals before making recommendations to approve or reject them. The applications are processed within 15 business days from the date of application, inclusive of physical verification of the site. If shortcomings are found, additional 30 days are given to the facility to rectify them [3].

PM-JAY incentivizes enlisted hospitals to continuously improve their quality of care, with bonuses provided for National Accreditation Board for Healthcare Providers (NABH) accreditation, teaching institutions, and hospitals in underserved areas. Additionally, there are tertiary and specialized care hospitals that operate as autonomous institutes of excellence directly under the Ministry of Health and Family Welfare, such as All India Institute of Medical Sciences (AIIMS), New Delhi and Jawaharlal Institute of Postgraduate Medical Education and Research (JIPMER), Pondicherry. These hospitals have been directly enrolled by the National Health Authority (NHA) through a Memorandum of Understanding (MoU), along with all NABH-accredited private hospitals in the National Capital Region. The enlisting of government hospitals is also underway to widen the network of service providers.

Role during the COVID-19 pandemic

National government-funded schemes are critical during times of public health emergencies, and it was fortunate for the people of India that the groundwork for such a nationalized health scheme had been laid before the pandemic. During the COVID-19 pandemic, PM-JAY provided financial protection to approximately 23 million individuals, covering over 160,000 hospitalizations related to COVID-19 under the scheme, leading to savings of over 1,800 crore Indian National Rupee (INR) (>260 million US dollars) for the beneficiaries and their families, according to the NHA [6].

PM-JAY has enrolled over 28,350 hospitals and healthcare providers, benefiting more than 519 million vulnerable families across India (as of May 31, 2023) [7]. Furthermore, the Ayushman Bharat scheme has established over 12 lakh (~1.2 million) health and wellness centers to provide primary healthcare services to people residing in remote and rural areas.

PM-JAY introduced COVID-19-specific packages in May 2020 that covered the costs of COVID-19 testing and treatment for beneficiaries, including expenses related to hospitalization such as room charges, nursing charges, Personal Protective Equipment (PPE) kits, doctor's fees, and other charges. The scheme has covered over 1.6 lakh (160,000) hospitalizations related to COVID-19 as of March 2021 [6].

PM-JAY provides emergency transport services through the National Ambulance Service (NAS), which includes more than 3,200 ambulances across India. Over 1.5 lakh emergency transport services were provided under PM-JAY in March 2021 [1].

To promote social distancing, PM-JAY has encouraged the use of telemedicine services during the pandemic. In March 2021, more than 1.6 lakh teleconsultations were conducted under PM-JAY, and the Ministry of Health and Family Welfare launched the eSanjeevani telemedicine platform in April 2020, which has conducted over 1.1 crore (~10 million) teleconsultations as of March 2021 [6].

Auxiliary pilot programs to strengthen healthcare delivery

Mohalla clinics have been introduced to address the issue of limited access to primary healthcare in the national capital state of New Delhi and the state of Punjab. These clinics, which derive their name from the Hindi word for "neighborhood" or "community," provide essential health services, including medicines, diagnostics, and consultation, free of cost. They serve as the first point of contact for the population, offering timely services and reducing the number of referrals to secondary and tertiary health facilities [8].

While the Mohalla clinic initiative, launched in 2015, has been a significant step towards improving healthcare access, it is important to note that these clinics do not fully encompass the concept of primary healthcare (PHC). PHC is a comprehensive approach that includes a balanced mix of preventive, promotive, curative, diagnostic, and rehabilitative services [8].

Mohalla clinics face several challenges in delivering effective primary healthcare services, including limited resources and services, lack of proper infrastructure, and lack of reliability, accountability, and awareness among the population. Limited resources and infrastructure can lead to delays in seeking medical care and may deter patients from seeking care altogether. This may result in patients seeking care from unlicensed or unqualified providers, leading to poor health outcomes. Additionally, a lack of awareness may lead to underutilization of Mohalla clinics' services. These challenges highlight the need for a comprehensive approach to primary health care that addresses the broader determinants of health, including infrastructure, resources, and awareness, in addition to clinical care. Collaboration between healthcare providers, policymakers, and the broader community is necessary to ensure that all individuals have access to the healthcare services they need to live healthy and productive lives [9]. This principle may apply to global healthcare, especially in resource-limited settings.

Challenges and ways forward

Ayushman Bharat Pradhan Mantri Jan Arogya scheme was launched in September 2018, and India has made progress towards Universal Health Coverage since. Although the start has been encouraging, there have been some challenges. Over the course of the coronavirus pandemic, it was evident that the nation is poorly prepared for challenges like these. Some of the issues that need to be addressed are the gap between supply and demand and increased government spending on healthcare. There is also a shortage of healthcare professionals in rural healthcare centers. Although quality medical care is available in urban areas, it can be difficult to access due to cost, distance, and dysfunctional systems. Therefore, it is imperative to address the doctor-patient ratio, especially in rural areas [10].

As mentioned above, 62% of India's total healthcare expenditure is out-of-pocket expenses which is one of the highest in the world. As a result, many families apply for personal bankruptcy as a result of medical debt. Financial support with avenues like medical crowdfunding can help with the process of fundraising. However, it is still in the nascent stages in India and will need the right awareness campaigns and training to bring a change in people's lives [10].

Given that the Ayushman scheme covers only inpatient illness and healthcare expenses around the period, this is more of a reactive measure than a proactive one. With a large population that has no regular healthcare access or focus on health education, a reactive scheme means more expenses and decreased outcomes. 

More healthcare schemes focusing on the prevention and early diagnosis of various diseases are required. This will not only help reduce the cost of healthcare substantially but also help increase productivity and the overall health of the nation. Integration of broader criteria, including visits to physicians, outpatient tests, and treatments, should be included. It should also be integrated with other regional schemes that cover other areas of healthcare which are currently outside the scope of Ayushman Yojna. Incentivizing patients with increased medical expenditure slabs could be considered for various categories (the current maximum limit is 500,000 Indian Rupees per family annually) [1]. For example, those who get annual or semiannual exams, who quit smoking or drinking alcohol, and those that do not smoke or drink alcohol and have optimal BMI. Many states do not recognize the scheme of the central government. Some have their own schemes which cover the population in a similar fashion but with some differences in reimbursement. It would be good to integrate this scheme with those and make a single scheme that applies to all parts of the nation without making it optional for states to implement. That way, health expenditure can be streamlined, uniformly implemented, and some expenses can be cut to a certain extent. There are various categories of people (individuals) who can not avail benefits of this scheme, such as those who own vehicles or if the monthly salary is more than INR 10,000, to name a few. Some of these are very basic day-to-day requirements and are met easily, but the person or family still needs support from the government. These should be included, and maybe the cutoff be implied based on higher monthly income rather than including someone who has a refrigerator in their house, which could have been gifted and is common in very poor households too, but this person might not have means to afford regular health care [11,12].

Hospitals sometimes prioritize cheaper products without ensuring quality, and early discharge of patients to reduce costs can lead to increased complications. Accessibility to biometric enrolment for scheme benefits is limited, causing inconvenience and potential harm to immobile or critically ill patients. Reimbursement issues arise due to insufficient or delayed payments, discouraging private institutes from participating. Inappropriate claim refusals by insurance officers also result in time-consuming disputes. Implementing fair and timely reimbursement mechanisms, providing mobile stations for biometric enrolment, and involving physicians in claim review can address these issues.

A significant portion of beneficiaries are unaware of this program, so the government's effort of advertising it over television, radio, and social media could play a crucial role to meet the mission of the program [13].

Conclusions

India has taken significant strides toward universal health coverage with their Ayushman Bharat initiative, providing comprehensive healthcare access across urban and rural regions of India. A proactive strategy was undertaken in Ayushman Bharat to combat disparate availability, access, and quality across rural regions in India. This program has had an immediate effect in improving healthcare access and financial protection while simultaneously encouraging infrastructure development for healthcare. This could be a role model for equitable global healthcare. Furthermore, this plan proved its worth during the COVID-19 pandemic by providing financial protection to millions of individuals. Although progress has been impressive, substantial challenges must be met - the gap between the supply and demand of healthcare services, increased government expenditure on health, and underfunded rural health centers. As part of an effective plan forward, increasing attention must be focused on prevention and early diagnosis, linking the Ayushman scheme with other regional schemes, and undertaking public awareness campaigns to maximize usage by its target beneficiaries. By meeting these challenges head-on, Ayushman Bharat can achieve its aim of offering equitable and cost-effective healthcare services for all Indians - fulfilling its role of contributing towards reaching universal health coverage as one of the sustainable development goals.
